# Investigating the quality of family planning counselling as part of routine antenatal care and its effect on intended postpartum contraceptive method choice among women in Nepal

**DOI:** 10.1186/s12905-020-00904-y

**Published:** 2020-02-18

**Authors:** Mahesh C. Puri, Matthew Moroni, Erin Pearson, Elina Pradhan, Iqbal H. Shah

**Affiliations:** 1 Center for Research on Environment, Health and Population Activities (CREHPA), Kathmandu, Nepal; 2 Department of Sociology, Development Studies, Lund University, Lund, Sweden; 3 Ipas, Chapel Hill, NC USA; 4 The World Bank Group, Washington, DC USA; 5 Harvard T. H. Chan School of Public Health, Boston, MA USA

**Keywords:** Quality of care, Post-partum family planning, IUD, Antenatal care, Contraceptive counselling, Nepal

## Abstract

**Background:**

Though modern contraceptive use among married women in Nepal has increased from 26% in 1996 to 43% in 2016, it remains low among postpartum women. Integration of counselling on family planning (FP) at the time of antenatal care (ANC) and delivery has the potential to increase post-partum contraceptive use. This study investigates the quality of FP counselling services provided during ANC visits and women’s perceptions of its effectiveness in assisting them to make a post-partum family planning (PPFP) decision.

**Methods:**

In-depth interviews (IDIs) were conducted with 24 pregnant women who had attended at least two ANC visits in one of the six public hospitals that had received an intervention that sought to integrate FP counselling in maternity care services and introduce postpartum intrauterine device insertion in the immediate postpartum period. IDIs data were collected as part of a process evaluation of this intervention. Women were selected using maximum variation sampling to represent different socio-demographic characteristics. IDIs were audio recorded, transcribed verbatim in Nepali, and translated into English. Data were organized using Bruce-Jain quality of care framework and analyzed thematically.

**Results:**

Overall, the quality of FP counselling during ANC was unsatisfactory based on patient expectations and experience of interactions with providers, as well as FP methods offered. Despite their interest, most women reported that they did not receive thorough information about FP, and about a third of them said that they did not receive any counselling services on PPFP. Reasons for dissatisfaction with counselling services included very crowded environment, short time with the provider, non-availability of provider, long waiting times, limited number of days for ANC services, and lack of comprehensive FP-related information, education and counselling (IEC) materials. Women visiting hospitals with a dedicated FP counselor reported higher quality of FP counselling.

**Conclusions:**

There is an urgent need to re-visit the format of counselling on PPFP during ANC visits, corresponding IEC materials, counselling setting, and to strengthen availability and interaction with providers in order to improve quality, experience and satisfaction with FP counselling during ANC visits. Improvements in infrastructure and human resources are also needed to adequately meet women’s needs.

## Background

Family planning (FP) has been a longstanding strategy of the Government of Nepal to ensure reproductive rights of women and families to meet their desired family size with healthy birth-spacing, and to promote the development of an educated and healthy population [1]. To achieve this, ambitious goals have been set, aimed at increasing access to voluntary FP services with a focus on poor, vulnerable and marginalized populations [1]. As a result, contraceptive prevalence of modern contraception among married women increased from 26% in 1996 to 43% in 2016 and the fertility rate fell from 4.6 children per woman in 1996 to 2.3 in 2016 [2]. Despite these gains, the unmet need for contraceptives remains very high with about 1 in 4 women reporting that they were not using contraception even though they do not want to become pregnant [2]. On average, Nepali women had 0.6 more children than they wanted [2] and 50% of all pregnancies in 2014 were unintended. 31% of all pregnancies in 2014 ended in an induced abortion and 13% in unplanned births [3]. In addition, the use of modern methods of contraception among married women has stalled at 43% in the last 10 years—the method mix in 2016 was female sterilisation (14.7%), injectables (8.9%), male sterilisation (5.5%), oral pills (4.6%), condoms (4.2%), implants (3.3%), and intra-uterine device (IUD) (1.4%) [2].

Though 57% of women reported receiving postnatal check-up in the first two days after the birth, most of them are unlikely to receive family planning counselling during post-partum check-ups [2]. Only 13% of women who had a live birth reported receiving information on family planning during the postpartum period [2]. Availability and capacity of service providers, availability of supplies, social and cultural beliefs, and access to services in remote areas are major factors impeding FP counselling.

The antenatal and immediate postpartum period is a time during which couples generally have multiple encounters with the health care system. Therefore, it may be a cost-effective and efficient opportunity to provide FP services that would not require additional staff, supervision or infrastructure [4]. The role of FP counselling during pregnancy, after giving birth, and after an abortion is to support the choice of the most suitable method of family planning and to resolve any issues that may arise with the selected method. If a woman, preferably with her partner, is able to make an informed choice, she is more likely to be satisfied with the chosen method and continue its use [5, 6].

Previous studies have documented many barriers that have prevented the use of family planning methods resulting in unplanned pregnancies [4].. Many families overlook post-partum contraception due to sociocultural issues such as lack of communication on family planning methods, the pervasive mistrust and fear that IUD can cause infertility, and methods not being suitable for women who still want to have children [7, 8]. Additional factors such as geographical and financial access, provider bias, poor method choice availability, lower status of women, medico legal restrictions, and fear of side effects act as barriers to postpartum family planning use [9].

Despite various advantages of IUD as well as government efforts to strengthen family planning counselling and increased availability of long acting FP methods, IUD use is very low in Nepal.

In 2015, the International Federation of Gynecology and Obstetrics (FIGO) launched an initiative for the institutionalization of postpartum family planning services, with a focus on PPIUD, as a routine part of antenatal counselling and delivery room services in six countries including Nepal. In Nepal, at least four antenatal care visits is recommended and 84% of women received antenatal care from a skilled provider [2]. The FIGO initiative involved counselling of women during visits for antenatal care, developing and distributing a brochure about family planning methods, wall chart, and display of family planning video messaging on televisions in waiting area. This initiative also included training of health care providers, and provision of IUD during the immediate postpartum period as an option to meet women’s contraceptive needs and preferences. The present study is part of the process evaluation linked to the large-scale evaluation of this intervention.

The purpose of this study is to understand the experiences and perspectives of women on FP counselling received during ANC visits, as well as the effectiveness of FP counselling to aid women to make informed PPFP decisions, including PPIUD. This study provides context to the findings in Pradhan et al. (2019), which showed that the intervention increased PPIUD counselling by 25 percentage points and PPIUD uptake by four percentage points. The same paper also found that if all women were counselled by the program, PPIUD uptake would have increased by 17 percentage points [10]. Another paper that examined provider perspectives on the FIGO-NESOG intervention in Nepal finds that the providers in the study hospitals were positive about implementing PPFP counselling and PPIUD insertion services as a part of routine maternity care, but identified several supply-side issues preventing them from providing high-quality care including: (1) shortage of human resources, (2) lack of supplies and (3) lack of support from the hospital management [11].

## Data and methods

This qualitative study is part of a large-scale impact evaluation study that used a hospital level stepped wedge cluster design. The evaluation sought to determine the effect of a program to PPIUD uptake in six public hospitals of Nepal (Bharatpur Hospital, Bheri Zonal Hospital, Lumbini Zonal Hospital, Koshi Zonal Hospital, BP Koirala Institute of Health Sciences, and Western Regional Hospital).

Group 1 hospitals started their intervention in the fourth month after baseline data were collected for a period of three months, while Group 2 hospitals started their program in the tenth month after baseline data were collected for a period of nine months. The intervention specifically comprised of training maternity care providers in postpartum family planning counselling, PPIUD insertion techniques, and complication management as well as the provision of information regarding family planning to hospital staff. The intervention program was implemented by the International Federation of Obstetricians and Gynaecologists (FIGO) in partnership with the Nepal Society of Obstetricians and Gynecologists (NESOG).

Facility staff were trained in FP counselling including PPIUD by the FIGO/NESOG intervention. Following the intervention period, in-depth interviews were conducted with 24 pregnant women after they received at least two antenatal counselling sessions but prior to delivery. Four women from each of the six facilities participating in the study were selected using maximum variation sampling to represent different demographic and socioeconomic groups based on income, education level (6 years of education or below and 7 years of education or above), and age (age 17 to 25 and those over 30 years of age).

The in-depth interview guide was first developed in English and translated into Nepali. The guide was pre-tested with similar women in a hospital in Kathmandu and finalized based on the pre-test results. Interview questions were grounded in topics related to care experience and interactions with providers in order to understand how women perceived the quality of counselling as well as the effectiveness of the intervention to assist women in making informed PPFP decisions. The interview question asked of women and providers about the “counselling” received/provided (translated into Nepali) and participants interpreted this for themselves. We have used the term “counselling” throughout to discuss what women and providers considered and do not imply this to be a two-way communication. Topics included: background profile of participants, contents of counselling, prior awareness and use of contraception, intended postpartum method selection and effectiveness of counselling services in motivating selection. Due to timing differences in rollout of the intervention between hospital groups, interviews were conducted over an 11-month period between April 2016 and March 2017.

The study was approved by Nepal Health Research Council. Informed consent was obtained from all participants. In-depth interviews were conducted by a female Nepali researcher in the language the participants felt most comfortable. A private room at each facility or nearby location was used for obtaining consent and conducting interviews. Interviews lasted 55 min on average. With permission from participants, all interviews were audio-recorded, transcribed word-for-word and translated into English for analysis.

Thematic analysis was employed to understand women’s experiences with the intervention and quality of the counseling they received. The Bruce-Jain framework was used to assess quality of care from client perspectives in our analysis, and results are presented by the six parts of the quality framework: choice of methods, information given to clients, technical competence, interpersonal relations, follow-up and continuity of care, and the appropriate constellation of services [12]. For this purpose, a preliminary codebook was developed based on the interview questions and initial reading of interview transcripts. Two coders independently reviewed transcripts line-by-line to generate initial codes and through collaboration, modified to develop the preliminary codebook. Transcripts were then repeatedly read and coded for content. Content codes were then grouped into thematic categories corresponding with themes of the larger study related to quality of care and effectiveness of the intervention, including provision and results of counselling. Key quotes that exemplified major study themes and emergent themes are presented in the results. Computer software ATLAS.ti (version 7.0) was used for organizing the text and attaching codes.

## Results

### Profile of participants

Women interviewed ranged in age from 17 to 34 years, all were pregnant at the time of interview, and 12 out of 24 women had no child yet born at the time of the interview. The majority (17 out of 24) were living in urban settings. Most women (20 out of 24) had completed secondary level education (10th grade) and most of them (13 out of 24) were homemakers; others worked in small shops or were daily-wage labourers, policewomen, or teachers. Interviewed women were mixed in terms of caste/ethnic composition. For example, 10 of 24 women belonged to upper caste groups, followed by relatively advantaged indigenous groups, so-called “untouchable” groups, and disadvantaged indigenous groups. Majority of women (17 women) were living in a nuclear family and had attended more than three ANC visits (Table 1). When compared to Nepal DHS 2016, our participants were slightly more urban and more educated. For example, In Nepal DHS 2016, 62.8% women were living in Urban and 50% had achieved secondary or higher level of education and 33% were not employed in the last 12 months [2].

**Table 1:** Selected characteristics of interviewed women

Current age (in years)	Number of women
17–24	12
25–34	12
Number of living children	
0	12
1	9
2	3
Ethnicity/caste	
Upper caste group	10
Relatively advantaged indigenous groups	5
Untouchable group (*Dalit)*	4
Disadvantaged indigenous group	4
Religious minority	1
Level of education	
Illiterate	1
Primary	3
Secondary	15
Above secondary	5
Main occupation	
Home maker	13
Shopkeeper	4
Daily wage labor	2
Teacher/police	5
Place of residence	
Urban	17
Rural	7
Household living condition	
Nuclear Family	17
Extended Family	7
Previous experience of using any modern family planning methods	
Yes	11
No	13
Previous experience of using IUD	
Yes	1
No	23
Number of ANC visits	
2	7
3	11
4 and above	6
Total	**24**

### Choice of methods

PPIUD insertion in the immediate postpartum period was a newly offered service through the intervention, and women were expected to be counseled on PPIUD in addition to other PPFP options during ANC. When FP methods counselling was provided during ANC visits, a single or few methods were mentioned, typically PPIUD, while further information about other methods was often gained from the hospital televisions or the FP brochure. For example:

*“They just provided information about IUCD here at … (name of hospital). But outside in the T. V, it provided information on IUCD, condom and I don’t remember other”.*

- ID 13, 17-24 years old, 10 years of education

The majority of women had PPIUD mentioned to them during ANC visits, but only about half stated that they were counseled on the method. Some women were told that they could receive PPIUD after delivery but were not given information that differentiated the option from PPFP methods.

*“They didn’t explain me everything about the method (PPIUD). They just told me that if I give birth to my child in that hospital and if I agree in using it, they will insert the Copper-T after I deliver my baby. They also stated that I can use it as long as I want to and can remove it if I do not have the desire to continue the method”.*

– ID 14, 17-24 years old, 2 years of education

In addition to counseling being limited to certain methods, women’s choice was limited due to their lack of decision-making power. Eleven out of 24 women were undecided with no clear relationship with their satisfaction with counseling and method selection. The primary decision maker among the majority of women were not themselves but their husbands. Some women explained that their husband’s wishes overrode their own, citing the pressure to have more children or continue childbearing until a boy is born. Intervention counseling focused on the woman herself, and in these cases, the woman did not have a full choice of methods despite their availability.

*“My husband takes the decision. According to his decision, we use the methods.*

*Personally, I want to use permanent methods. For that I need to consult with my husband, but my husband won’t agree to it. If only I get a son this time, he might agree otherwise he won’t”*

– ID 12, 17-24 years old, no formal education

### Information given to clients

#### Counseling

Among the 24 women interviewed, 11 (9 in Group 1 hospitals and 2 in Group 2 hospitals) reported not having received family planning counselling. Women who received some information expressed a feeling of not having been counseled at all due to brief appointments with incomplete information contrary to what was expected (Table 2).

*“If I am counseled and if I understood better about the temporary methods, maybe I will use temporary method. But I have not understood much about the methods. I may use the temporary method once I understand properly and after consultation”.*

- ID 16, 25-34 years old, 10 years of education

When counselling was received, it was often seen as insufficient in aiding women’s decision-making process:

*“I didn’t receive the counselling that I should have received from the health workers. So, the counseling that I had received hasn’t influenced me. Maybe in upcoming visits if I receive counselling then it will influence me”.*

– ID 19, 25-34 years old, 12 years of education

*“I haven’t taken any decision till now because I don’t have any information about the contraceptive methods. No one has told me about them. Since I don’t know about them, I haven’t made a decision yet”.*

– ID 9, 17-24 years old, 2 years of education

Limited counselling and lack of adequate information from healthcare providers led women to depend on information from friends, and family rather than counseling received through ANC care. While often accurate, some women shared incorrect information received from such networks.

*“My friends, elder ones told us that it (IUD) is difficult if we use it. They told that it is better to take injections than using this”.*

– ID 17, 17-24 years old, 6 years of education

*“She also told me that I can get pregnant if I don’t use contraceptive or we can use withdrawal method. But some men don’t agree so they don’t use withdrawal method but if my husband ejaculates inside then she suggested me to go to urinate immediately and that it will come out”.*

– ID 11, 17-24 years old, 12 years of education

For some women, it was information from the negative experiences of others that guided their decisions due to lack of or incomplete and poor counseling during ANC.

*“I am scared to use copper-T because my uncle’s wife had used this and after one year of its insertion she started having stomach pain and she was brought to Metro City at Srijana chowk to take out the IUCD and then only her pain stopped. Also, it is made of copper so I am scared to use that”.*

– ID 04, 17-24 years old, 10 years of education

*“Simply, I just did not like them. My sister had used it but that did not suit her and she had heavy bleeding. She was about to die so because of this incident my husband fears and does not allow me to use any contraceptives. I also fear if same thing will happen to me”.*

*– ID 21, 25-34 years old, 5 years of education*

**Table 2:** Women’s response on whether received counselling, FP brochure and satisfaction during antenatal care visits by type of hospitals

ID	Whether received FP Counselling	Whether received FP Brochure	Whether brochure explained	Whether information provided about PPIUD	Type of provider who counselled	Counselled individually or provided FP education in group	Whether satisfied	Type of method chosen
Group 1 hospitals								
1	No	Yes	No	No	–	–	Yes	Implant
2	Yes	Yes	No	Yes	/Doctor	Group	No	Condom
3	Yes	Yes	Yes	No	Nursing student	Group	No	Sterilization
4	Yes	Yes	Yes	Yes	Nurse	Group	No	Implant
5	No	No	–	No	–	–	No	IUD
6	No	No	–	No	–	–	No	Not decided
7	No	Yes	No	No	–	–	No	Not decided
8	Yes	Yes	Yes	Yes	Nursing students	Individual	Neutral	Not decided
9	No	No	–	No	–	–	No	Not decided
10	No	Yes	No	No	–	–	No	Sterilization
11	No	No	–	No	–	–	No	Injectable
12	No	No	–	No	–	–	No	Injectable
Group 2 hospitals								
13	Yes	No	–	Yes	Doctor	Group	No	PPIUD
14	Yes	Yes	Yes	Yes	Nursing student	Group	No	Not decided
15	No	Yes	Yes	No	–	–	No	Not decided
16	No	No	–	No	–	–	No	Not decided
17	Yes	Yes	Yes	Yes	Nurse	Group	Yes	Not decided
18	Yes	Yes	Yes	Yes	Nurse	Group	Yes	Not decided
19	No	Yes	No	Yes	–	–	No	Withdrawal
20	Yes	Yes	No	Yes	Nurse	Group	Yes	Not decided
21	Yes	Yes	Yes	Yes	Nurse	Individual	Yes	Withdrawal
22	Yes	Yes	No	Yes	Nurse	Individual	Yes	Sterilization
23	Yes	Yes	No	Yes	Nurse	Individual	Yes	Not decided
24	Yes	Yes	Yes	Yes	Nurse	Individual	Yes	PPIUD

#### Brochure

When asked whether women received FP brochure and if the content was explained to them, most women (17 of 24) reported that they received the FP brochure and only a little more than half (9 out of 17) reported that the providers explained the content in the brochure (Table 2). The other half did not have the contents of the brochure explained to them, instead being told to read it at a later time. One such woman said:

*“They didn’t explain well about any of the methods. They just gave this card and provided general information only. They just said to read the card at home. Maybe they will provide information in upcoming visits”.*

- ID 19, 25-34 years old, 12 years of education

However, the women who were counseled and received a copy of the FP brochure said the brochure reinforced the information they had already received from providers. In these cases, the brochure was seen as a positive resource. For example, one participant said:

*“I liked it. If one doesn’t understand when explained in the hospital, one can read thoroughly at home. There were pictures of all devices and they also explained by showing the pictures.”*

- ID 24, 17-24 years old, Bachelor’s Degree level of education

Women who did not receive counseling were confused about the purpose of the brochure, and uncertain about when they would receive the full information on family planning. For example, one woman said:

*“They gave me the card, but they didn’t tell me what was written on the card. They should have counseled me while providing the card and I would have understood about it. How will I know without them telling me?”*

- ID 09, 17-24 years old, 2 years of education

A significant barrier to women learning about the methods from the brochure that emerged during interviews was other responsibilities taking priority over the possibility of reading the FP methods brochure at a later time.

*“Since I have to do labor work, I haven’t got time to read it. I get tired when I come back home at night and instead of reading I feel like sleeping so I haven’t read that.”*

– ID 01, 17-24 years old, 7 years of education

Literary and language also affected the usefulness as the brochure was only available in Nepali. Some women expressed that the brochure was not helpful to them if they could not read.

*“Many Madhesis and Muslims women are illiterate, so for those women the brochure is not helpful. Rather than providing brochure to those women, it would be better if they provide verbal counselling when they visit for antenatal checkup. Those who can read can understand after reading it so for them the card is useful but it wouldn’t be useful for uneducated women”.*

- ID 15, 25-34 years old, Bachelor’s degree level of education

#### Video

The video was also produced only in Nepali, and the video content did not benefit women who could not understand the audio or subtitles in Nepali. Some women cited problems of overcrowding and high noise volumes in the waiting room limiting their ability to see and hear the information provided on televisions. One participant explained:

*“When I watched, I used to read what was written on the screen and sometimes I didn’t read. It was too noisy and the sound was also low. So, I couldn’t hear properly which methods are available”.*

– ID 11, 17-24 years old, 12 years of education

However, for those who were able to see, hear and understand the video content, some cited its benefits outweighing that of the limited or lack of information received from providers. A few women explained that information played or displayed in hospitals were more helpful to their decision-making than counselling:

*“More than today’s counselling I learned from information provided through TV, and I think I should use any one of the method till the time my husband uses permanent method”.*

– ID 03, 25-34 years old, Master’s degree level of education

### Technical competence

Women who received counseling on PPFP were mostly satisfied with the technical competence of their providers to give trustworthy information. Noted satisfaction was highest among Group 2 hospitals women who explained that they had gained new information about FP methods from providers. For example:

*“I received a lot of knowledge. Women might not have knowledge on which method to use after the delivery of the child. So, after receiving such counselling, they will have knowledge on which method to use. So those who are unaware will have knowledge about it. Even I didn’t know that we can use copper-T right after delivery, I only knew that it is a temporary method. I only came to know that it can be used right after delivery after receiving counselling”.*

– ID 23, 25-34 years old, 10 years of education

Women expressed that they trusted the opinions of doctors most over the views of friends and family, although care was primarily provided by nurses.

*“They will be having good information. They will have knowledge about what methods we adopt have what benefits. It is better to adopt the method after discussion with doctors than blindly believing in what others say.*

– ID 05, 17-24 years old, 9 years of education

### Interpersonal relations

Most women (15 out of 24) were not satisfied with the care they received, noting that they did not receive proper information and were often hurried through their appointments due to crowding and resource shortages at hospitals (Table 2). A woman shared her experiences as follows:

*“The doctor checked my belly and said that everything was fine. She didn’t ask why I came and what had happened. She didn’t even ask if I had any queries or wanted to know anything. She just rushed the check up and let me go”.*

– ID 13, 17-24 years old, 10 years of education

A barrier to positive interaction with providers regularly communicated by women was the crowded setting of hospitals, leading to limited time with providers, ranging from few to fifteen minutes.

*“It would have been better if I had the opportunity to spend the time like I am spending with you (researcher) but I couldn’t talk for long with them (service provider). I think we didn’t even spend 5 minutes”.*

– ID 19, 25-34 years old, 12 years of education

Long waiting times and rushed nature of visits led to a perceived inability to engage in dialogue or ask questions, and a feeling of lacking information to make informed PPFP decisions. One woman explained:

*“Only she spoke, and I listened to her. She just provided information about copper-T that too she said it in a rush. She didn’t ask me anything else; she didn’t even ask whether I understood what she had said. I hadn’t understood well about copper-T and I wasn’t even given a chance put any queries”.*

– ID 13, 17-24 years old, 10 years of education

The dominant sentiment among the majority of women was that providers were often too busy, resulting in rushed appointments and poor attitudes or angry reactions when women inquired about their care. For example,

*“They are in a rush and do not tell anything properly. If we ask them about when my turn will come, they get angry and scold us. They speak in a rude way and answer that if we want our turn to come early, we can go to the other hospitals”.*

– ID 14, 17-24 years old, 2 years of education

There was a perceived lack of accountability of staff; providers arriving after opening hours and leaving before closing time, resulting in a limited window of availability for women to be seen despite having travelled long distances in some cases.

*“ … they go for meal at 12 noon. Then they come back around 2:00 and after seeking the patients for 1-2 hours they say to visit tomorrow or the next week. The one who live nearby will arrive early. But for the one living far it is hard”.*

– ID 09, 17-24 years old, 2 years of education

*“The sisters in the hospital do not do anything. They don’t give time to ask any question and do not listen to us. They are in a hurry and you cannot find anyone for pregnancy check after 12pm. Since they do not listen to us properly, how am I supposed to ask them? I listened to whatever they said and returned home’.*

– ID 14, 17-24 years old, 2 years of education

For most women, their experiences resulted in feelings of not having been counseled, insufficient level of care, and not having their concerns heard.

*“The doctors and nurses doesn’t pay attention to our concerns. It’s not just me; I didn’t find them counselling properly to other woman as well. I didn’t see them asking them if those women had any questions. I have seen that due to crowd, the counselling place was unmanaged so they couldn’t provide proper counselling to women”*

– ID 03, 25-34 years old, Master’s Degree level of education

However, some women were happy with their interactions with providers, noting that they received answers to their questions and that providers seemed to attempt to provide information when possible.

*“I felt good while talking to them. I was happy that they gave me these sorts of information. We knew little bit about these but for the ones who did not know about these methods, this counselling was very helpful to them”.*

– ID 22, 25-34 years old, 10 years of education

There were noted differences of women’s experience of ANC by hospital groups, with some women from Group 2 hospitals with designated counselor reporting a positive experience:

*“They treated me well. They told me to not get worried when they said that I have twins. They told me to use contraception method after the delivery of the child. Everyone was treated well, they told the same thing to others as well”.*

– ID 24, 17-24 years old, Bachelor’s degree level of education

### Appropriate constellation of services

The intervention sought to integrate PPFP counseling into ANC care, but the level of integration varied widely. While 13 out of 24 women reported having received FP counseling as part of ANC appointments, the level of care, depth and quality of information they received varied significantly, ranging from (i) basic checkup of weight measurement and blood pressure to (ii) limited counseling on ANC and PNC topics to (iii) in-depth information on FP methods, advantages and disadvantages, care of mother and baby, and delivery. When counseling was provided, it was most often from a nurse. PPFP counselling was highest in Group 2 hospitals, which provided FP counselling, brochures, explanation of brochures, and counselling to almost all women. Most women (19 out of 24) reported that they were provided FP education in a group (Table 2).

As expected, appointments often began with a basic checkup during which some women were asked about previous contraceptive use, given tetanus toxoid injections or instructed to obtain them from outside pharmacies. For some women, the appointment (and further appointments) did not progress further to include FP methods or other delivery-related topics despite perceptions that such care and advice would be provided throughout their pregnancy. For example, a woman explained:

*“I went for a checkup today morning and they just did my check up and didn’t say anything. They told me to come after either in one month or one and a half month. If they can tell us the ways to take care of ourselves, our baby, things which can do in pregnancy and things to avoid during pregnancy, things we should eat and things we shouldn’t eat, the complications that may arise in pregnancy etc, then it will be beneficial for uneducated women like us”.*

– ID 14, 17-24 years old, 2 years of education

## Discussion

By applying the Bruce-Jain quality of care framework, this paper investigates women’s perspectives and experience of the quality and effectiveness of FP counselling provided as part of antenatal care appointments in the six tertiary public hospitals in Nepal. Our findings suggest that most women received poor quality of counselling in the study hospitals as determined by the frequency and depth of information provided to them, technical competence of providers, and interpersonal relations between women and providers. Women often attributed poor quality of care to lack of organization and infrastructure. The 2018 revision of the Bruce-Jain framework for quality of care in rights-based family planning service provision demonstrates that the key facets of quality of care can be divided into structure and process [13], and this study finds that while problems exist at both levels, structural problems were the largest barrier to high quality service provision. Study findings suggest that it is difficult to successfully implement a PPFP intervention in tertiary care facilities without first addressing resource constraints in the health facilities. Women discussed basic barriers to receiving high quality care in some of the facilities such as overcrowding, too little time spent with providers, and long wait times.

We found that ANC appointments with providers are some of the few times women have to learn about their family planning choices as they have other pressing and competing priorities outside of facility visits. Responses indicate changes are needed to better structure ANC appointments and FP counselling in order to increase quality and impact. Shortage of staff and crowded environment of hospitals had a negative impact on the impressions of the majority of women, leading to perceived unsatisfactory experience and lack of information to make an informed decision that steered some women to continue with the FP method they used previously or rely on withdrawal. Some of these barriers could be alleviated by establishing an appointment booking system that prioritizes women by criteria such as need and distance travelled, limiting the number of women booked on a given day, and assigning specific time slots to limit crowding in hospital waiting areas.

In recent years, there is a movement to reinvent ANC, including by providing group ANC. A study conducted in Kenya and Nigeria shows that women who attended group ANC were more likely to deliver in a facility than women who attended routine individual ANC in Nigeria, but not in Kenya where facility delivery associated with individual care was equally high. In both countries, women enrolled in group ANC received higher quality ANC care than women in individual ANC [14]. Similarly, another study conducted in Ghana found that group ANC as compared to individual ANC offers an opportunity to increase quality of care and improve maternal and newborn outcomes including for intention to use postpartum FP [15]. This could be one of the approach that Nepal can think of strengthening in such high caseload health facilities.

Women reported that doctors were the most trusted source of information over nurses, suggesting that their inclusion in FP counselling could prove beneficial in absorbing information and improving perceptions about quality of care.

Overall, the quality of counselling was poor for many of the women interviewed. Most women reported receiving information on FP from family or elders rather than health providers. Social networks have a strong influence on women’s contraceptive use [16], and though providers, especially doctors, were a trusted source of information, women relied on their previous experience and information from social networks in the absence of adequate counselling, especially by doctors.

Most women in Group 2 hospitals received counselling on PPIUD, and this may be due to the fact that intervention was adapted and a designated counselor was posted in these study hospitals. Among the women who did receive postpartum family planning counselling as a part of their ANC visit, some were counseled only on PPIUD rather than a broad range of methods as the intervention intended. This is a challenge in health systems interventions that seek to add a service, as patients are sometimes offered only the newly added service. This could be due to requirements that providers practice the new service until they achieve proficiency, or it could result from more intense monitoring of the newly added service.

Finally, many women reported that their husbands were the primary decision-makers of family planning, which suggests that even with high quality counselling, women may not be able to use the method they wish. Interventions that include men, either through including them in women’s ANC counselling or in men-only counselling, could work to increase informed PPFP decisions [17–20]. Interventions that offer women a PPFP brochure to take home for discussion with their husbands have been used in other settings and could be more feasible than getting the husbands to come to the clinic for in-person counseling [21]. The brochure could be given at the first ANC visit, with more counseling provided at subsequent ANC visits after the woman has discussed the brochure with her husband.

Video content played on waiting room televisions seemed to be a promising approach to counselling when providers did not have time to provide in-depth counselling, and it also avoids issues of bias in counselling in that it presented all methods rather than just the new PPIUD service being offered. In some hospitals, the video content that played on facility televisions served as the only source of FP information, and some women thought that the video was more beneficial than the counselling they received. However, some women reported difficulty seeing and hearing the video due to overcrowding in the hospital waiting area. Subtitles were included in the video, but this was not helpful for women with little or no literacy or when the screen was small or far away. Similarly, the FP brochures were not very helpful to most women in the absence of counselling, especially for low literacy women. The video had the added advantage of being played while women were waiting to see a provider, whereas the FP brochures were handed out at the end of the visit, leaving women to review them on their own time when they may have other competing priorities.

Findings presented in this study should be interpreted in light of the following limitations. Data were collected from a convenience sample of women who received at least two ANC visits, and pregnant at the time of interviews in the six hospitals participating in the FIGO/NESOG intervention. Therefore, findings may not generalize to women receiving ANC in other hospitals in Nepal. Nonetheless, our sample included women from diverse socio-demographic and cultural backgrounds hence providing perspectives on married women in Nepal. In addition, this study relies solely on self-reporting by women who received ANC care in study facilities. Reliance on self-reported information suffers from potential recall errors and reporting biases. The information presents women’s interpretation of provider’s behaviour and care they have received rather than an objective third-party assessment. A paper that documented providers perspectives showed that they were highly motivated to provide quality family planning FP services; however, they also identified several facility and training related factors which could prevent them from doing so [11].

Despite these limitations, this study documents women’s views on the quality of family planning counselling services in these six study facilities in Nepal. The study also documents that (i) FP brochure is useful mostly in conjunction with counselling for literate women, (ii) the FP video in the ANC waiting room could be useful for both literate and non-literate women, (iii) interaction with providers is often too short, in an environment that is too crowded and often disrespectful of women, with information that is inadequate for women to act on their family planning priorities, as a result of which (iv) women are mostly dissatisfied with the FP counselling service they have received, and (v) do not have all of the information required for PPFP decision-making.

## Conclusions

Provision of quality FP counselling during ANC visits is an important factor of PPFP acceptance for informed policy on improved maternal and child health in Nepal. Our study findings imply that improving PPFP uptake among women requires improvements in health system infrastructure (health provider availability, provider knowledge and behavior with clients) to allow for ample time and resources during ANC visits in order to first understand clients’ needs and preferences to (i) provide client-centered counseling on family planning methods taking into account each client’s particular circumstances to tailor information on relevant method options, (ii) strengthen use of PPFP IEC video playing in ANC waiting rooms to utilize the limited time women may have to gain information about family planning, and (iii) involve male partners in the family planning counseling sessions and discussions. Women’s narratives highlight that counseling to be effective, responsive and high quality would require it to be client-centered taking account their individual circumstances and preference. These qualitative findings have implications for improving family planning services in Nepal, particularly as part of ANC and PNC services in hospital settings.

## Data Availability

Once de-identified, the dataset used and/or analysed during the current study will be available from the corresponding author on reasonable request.

## Abbreviations

ANC: Antenatal care
CREHPA: Center for research environment health and population activities
FIGO: International Federation of Obstetricians and Gynaecologists
FP: Family Planning
IDI: In-depth interviews
IEC: Information communication and education
IUD: Intra-uterine device
NESOG: Nepal Society of Obstetricians and Gynaecologists (NESOG)
PNC: Post-natal care
PPFP: Post-partum family planning
PPIUD: Post-partum intra-uterine device
